# Environmental and socioeconomic analysis of malaria transmission in the Brazilian Amazon, 2010–2015

**DOI:** 10.11606/S1518-8787.2019053000983

**Published:** 2019-05-15

**Authors:** Tiago Canelas, Carlos Castillo-Salgado, Oswaldo Santos Baquero, Helena Ribeiro

**Affiliations:** I Universidade de São Paulo. Faculdade de Saúde Pública. Departamento de Saúde Ambiental. São Paulo, SP, Brasil; II Johns Hopkins University. Johns Hopkins Bloomberg School of Public Health. Baltimore, MD, US; III Johns Hopkins University. Johns Hopkins Bloomberg School of Public Health. Global Public Health Observatory. Baltimore, MD, US; IV Universidade de São Paulo. Faculdade de Medicina Veterinária e Zootecnia. Departamento de Medicina Veterinária Preventiva e Saúde Animal. São Paulo, SP, Brasil

**Keywords:** Malaria, epidemiology, Risk Factors, Socioeconomic Factors, Spatial Analysis, Amazonian Ecosystem, Health Status Disparities

## Abstract

**OBJECTIVE:**

To analyze the environmental and socioeconomic risk factors of malaria transmission at municipality level, from 2010 to 2015, in the Brazilian Amazon.

**METHODS:**

The municipalities were stratified into high, moderate, and low transmission based on the annual parasite incidence. A multinomial logistic regression that compared low with medium transmission and low with high transmission was performed. For each category, three models were analyzed: one only with socioeconomic risk factors (Gini index, illiteracy, number of mines and indigenous areas); a second with the environmental factors (forest coverage and length of the wet season); and a third with all covariates (full model).

**RESULTS:**

The full model showed the best performance. The most important risks factors for high transmission were Gini index, length of the wet season and illiteracy, OR 2.06 (95%CI 1.19–3.56), 1.73 (95%CI 1.19–2.51) and 1.10 (95%CI 1.03–1.17), respectively. The medium transmission showed a weaker influence of the risk factors, being illiteracy, forest coverage and indigenous areas statistically significant but with marginal influence.

**CONCLUSIONS:**

As a disease of poverty, the reduction in wealth inequalities and, therefore, health inequalities, could reduce the transmission considerably. Besides, environmental risk factors as length of the wet season should be considered in the planning, prevention and control. Municipality-level and fine-scale analysis should be done together to improve the knowledge of the local dynamics of transmission.

## INTRODUCTION

Malaria is still a major burden on the health in the world, in the Americas and in Brazil. Despite the significant progress of Brazil in controlling the disease since 2005, 193,670 malaria cases occurred in 2017, a 35% increase over the 143,459 cases in 2015 ^1^ . In 2015, the Brazilian National Malaria Control and Prevention Plan (NMCP) for 2003–2015 ^2^ was ended, a successful national plan that helped to achieve the Millennium Development Goals milestone of a 75% reduction in malaria cases from 2000 to 2015 ^3^ . In 2016, a new national strategy ( *Plano de Eliminação da Malária no Brasil* ), together with the Global Technical Strategy for Malaria, was launched to eliminate malaria by 2030 ^4^ . This is not the first time Brazil faces a sudden increase in the number of cases after a successful national strategy ^5^ ^,^ ^6^ . Therefore, understanding the local epidemiology and the risk factors enabling this increase is crucial.

The Brazilian Amazon is a huge territory with large heterogeneities, and studying the risk factors of malaria transmission for this whole area is a big challenge. In recent years, several studies analyzed the epidemiological profile of the entire Brazilian Amazon and found a reduction in the malaria distribution from 2005 onwards, but moderate or high transmission areas persisted^3,7–10^. Other studies assessed the socioeconomic and environmental risk factors at different scales and time periods with diverse findings in the influence of each risk factor^11–18^. Notwithstanding, there is a lack of studies investigating the risk factors, both environmental and socioeconomic, at municipality level, which is the lowest level the NMCP had operated in Brazil. Therefore, studying the risk factors at this level is crucial to plan interventions efficiently. Thus, our aim was to analyze the environmental and socioeconomic risk factors of malaria transmission at municipality level from 2010 to 2015 in the Brazilian Amazon.

## METHODS

### Study Site

**Figure 1:**
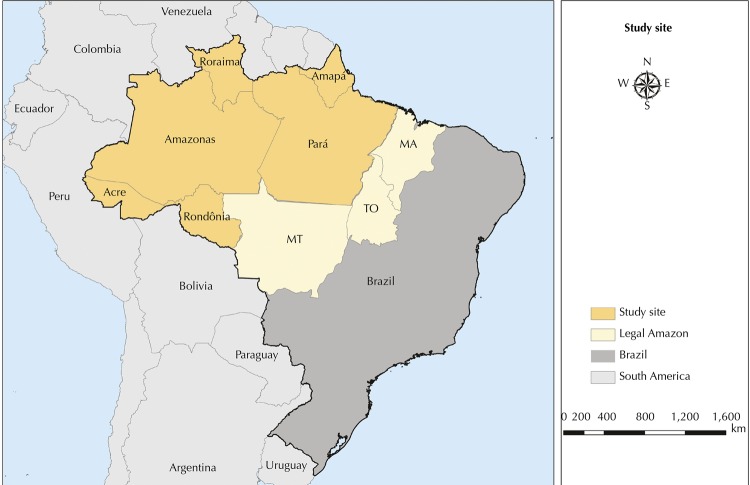
Map of the study site showing in dark yellow the states within the Brazilian Amazon forest from where we obtained the malaria data.

Source: Elaborated by the authors with data from IBGE.

The study site is located inside the Brazilian Amazon forest ( Figure 1 ), where 99% of the Brazilian cases are concentrated ^1^ . This area comprehends 311 municipalities in six states in the Northern Brazilian region. Our analysis excluded the municipality of Mojuí dos Campos because it was officially emancipated from Santarém in 2013, and no information is available for most of the years.

### Data

This is an ecological study, and we obtained the monthly autochthonous confirmed cases, from 2010 to 2015, from the system for epidemiological surveillance information (SIVEP/Malária). Since the aim was to understand malaria transmission within the Brazilian Amazon, we excluded the cases imported. We calculated the annual parasite incidence (API) (confirmed cases during 1 year / population under surveillance x 1,000) in each of the 310 municipalities. The API is the measurement Brazil uses for the municipality risk of malaria transmission. Yearly population data were obtained from the Brazilian Institute of Geography and Statistics (IBGE).

After exploratory analysis of several risk factors, and based on different studies, we selected the following covariates for each municipality for our model: Gini index, illiteracy, presence of legal mines, percentage of indigenous area, percentage of forest, and wet season length ( Box ). Gini index and illiteracy measure inequalities and are fixed values for the whole period since no official data is found for other years, and we consider the 2010 value a good proxy for the period 2011–2015. We selected the operative mines or those with work in progress for each year as they have been identified as a major problem for malaria transmission ^12^ ^,^ ^19^ . In Brazil, the law protects indigenous reserves, which are considered high risk areas ^2^ ; therefore, we calculated the percentage of the municipality that overlaps with the indigenous reserves in 2017. Forest coverage by municipality was obtained from *Projeto de Estimativa do Desflorestamento da Amazônia* (PRODES), which surveys the Amazon forest and provides yearly estimates ^20^ . To calculate the wet season length, we first extracted the monthly precipitation from the Tropical Rainfall Measuring Mission (TRMM) ^21^ by municipality, and then we calculated all the months with more than 100 mm ^3^ following the methodology by Valle et al. ^12^

**Box:** Year, source, unit and spatiotemporal resolution of the covariates included in the study.

Socioeconomic and Environmental Covariates					
Variable	Year	Source	Temporal res.	Spatial res.	Units

Gini index	2010	Census	Yearly	Municipality	Index
Illiteracy	2010	Census	Yearly	Municipality	%
Mines	2010–2015	DNPM	Yearly	Area	Number of operative mines
Indigenous area	2017	FUNAI	Yearly	Area	%
Forest coverage	2010–2015	PRODES	Yearly	Area	%
Length of the wet season	2010–2015	TRMM	Monthly	0.25° (28 km^2^)	Number of months

DNPM: *Departamento Nacional de Produção Mineral* ; FUNAI: *Fundação Nacional do Índio* ; PRODES: *Projeto de Estimativa do Desflorestamento da Amazônia* ; TRMM: Tropical Rainfall Measuring Mission

### Data Analysis

To assess the environmental and socioeconomic risk factors for malaria transmission, we stratified our dependent variable (median malaria API from 2010 to 2015) into three categories, following the stratum of the NMCP ^2^ . The strata were: API ≥ 50, high transmission; 10 ≤ API < 50, moderate transmission; and 1 ≤ API < 10, low transmission.

We created three models: the socioeconomic model, with the covariates Gini index, illiteracy, presence of mines and indigenous reserves; the environmental one, including forest coverage and wet season length; and the full model, including all the covariates.

To predict the differences in the influence of risk factors between low transmission municipalities and medium and high transmission municipalities, a multinomial logistic regression was performed for all three models using R 3.5.0 software. Maps and risk factors processing were done in ArcMap 10.5 (ESRI, Redlands, CA). The selection of the best model was based on the lowest Akaike Information Criterion (AIC).

## RESULTS

**Figure 2:**
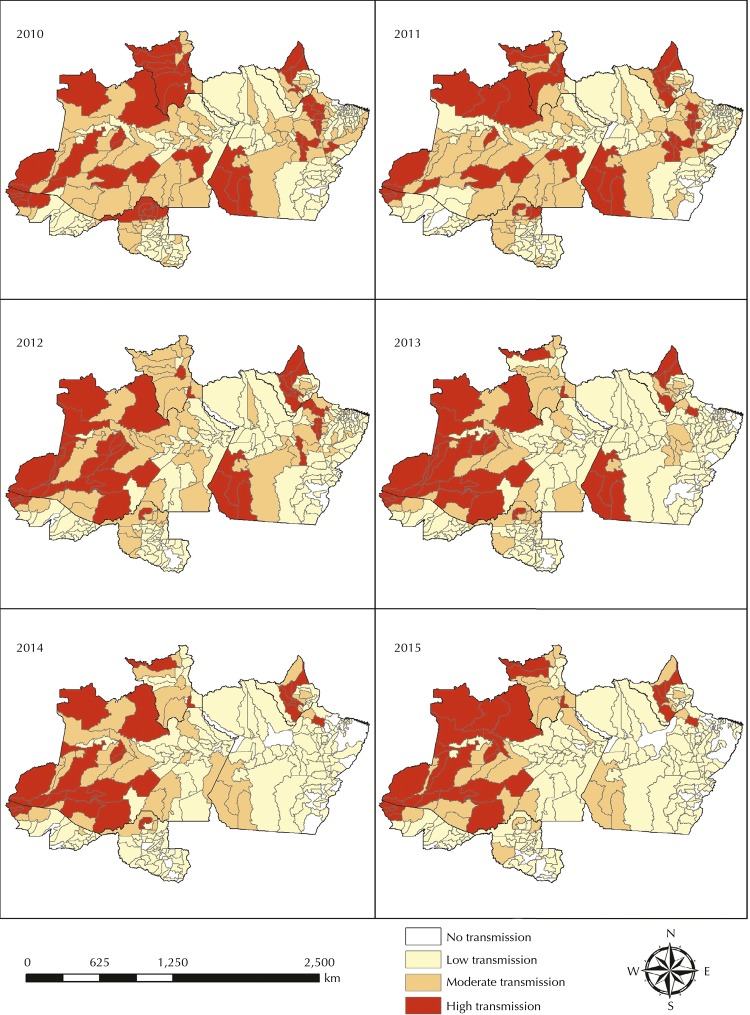
Maps of malaria transmission intensity from 2010–2015 in 310 municipalities within the Brazilian Amazon forest.

Source: SIVEP-malária^1^.

Figure 2 and Table 1 show the malaria distribution by intensity for the period 2010–2015. The number of high transmission municipalities declined over time, being, in 2015, concentrated mostly in the northwest (states of Amazonas and Acre) and in the state of Amapá. High transmission municipalities disappeared from Rondônia, and in the state of Pará, they only persisted in Anajás. An apparent concentration of municipalities with high transmission bordering other countries is also observed. Contrarily, an increase occurred in municipalities with permanent no transmission over the period.

Projection: DIRGAS 2000

**Table 1:** Number of municipalities by transmission intensity from 2010 to 2015.

Variable	2010	2011	2012	2013	2014	2015
High transmission	49	39	41	33	26	29
Moderate transmission	81	79	66	43	38	32
Low transmission	169	168	172	189	168	175
No transmission	11	24	31	45	78	74

Source: SIVEP-malária^1^.

Table 2 shows the variables descriptive statistics. It is worth mentioning the large variability in our dependent variable, API. Also, we found municipalities where the area of forest (Amazonas and Acre) or indigenous reserves (Normandia and Uiramuta, state of Roraima) range from none to almost the whole municipality. Similarly, there are municipalities in the state of Amazonas without dry season and others with a short 3-month wet season.

**Table 2:** Descriptive analysis of the covariates to be included in the models.

Variable	API	Gini index	Illiteracy	Mine	Indigenous area	Forest area	Wet season
Mean	21.61	0.59	16.85	35.98	11.93	44.69	8
Median	1.55	0.58	14.88	17	0.24	40.34	8
Maximum	914.66	0.81	40.06	1364	98.72	98.73	12
Minimum	0	0.43	3.33	0	0	0.12	3
Standard deviation	58.92	0.06	7.35	91.13	20.60	28.75	1.69

The results of the multinomial regressions are shown in Table 3 . The full model has the lowest AIC, indicating the best performance. When comparing high transmission municipalities with low transmission municipalities, the Gini index and the wet season length are the most important risk factors. As Gini index was scaled for the analysis, we expect to see a 106% increase in the odds of being high transmission per 1-unit standard-deviation increase in the Gini. For every month longer in the wet season, the API increased by 73% the odds to have high transmission. Thus, larger wealth inequalities and longer wet seasons are the main drivers of high transmission municipalities.

**Table 3:** Multinomial regression of the socioeconomic, environmental and full models.

Variable	Socioeconomic model	Environmental model	Full model
		
	OR (95%CI)	OR (95%CI)	OR (95%CI)
Medium/Low			
GINI index	1.57 (1.08–2.27)*		1.48 (0.99–2.19)
Illiteracy	1.08 (1.03–1.13)*		1.05 (1.00–1.10)*
Mines	1.01 (1.00–1.02)*		1.01 (1.00–1.02)*
Indigenous	1.02 (1.00–1.03)*		1.01 (0.99–1.03)
Forest		1.04 (1.02–1.05)*	1.03 (1.01–1.04)*
Dry season		1.11 (0.86–1.43)	1.18 (0.89–1.57)
High/Low			
GINI index	2.27 (1.38–3.73)*		2.06 (1.19–3.56)*
Illiteracy	1.15 (1.08–1.22)*		1.10 (1.03–1.17)*
Mines	1.01 (1.01–1.02)*		1.01 (1.00–1.05)*
Indigenous	1.01 (0.99–1.04)		1.00 (0.98–1.03)
Forest		1.05 (1.02–1.07)*	1.03 (1.00–1.05)*
Wet season		1.62 (1.18–2.22)*	1.73 (1.19–2.51)*
AIC	421.89	410.20	388.61

AIC: Akaike information criterion

* Statistically significant (p < 0.05).

All risk factors were statistically significant in the comparison between high and low transmission, except the percentage of indigenous area. In addition to Gini and wet season, for each unit of increase in illiteracy, we expect a 10% increase in the odds to have high transmission. The influence of mines and forest was very marginal.

However, when we compare medium transmission municipalities with low transmission municipalities, the overall influence of the risk factors is much weaker, and Gini index and wet season length are not statistically significant. Only illiteracy, the number of mines, and forest area are statistically significant. All three risk factors are very marginal, being illiteracy the one with the strongest influence OR = 1.05 (95%CI 1.00–1.10).

A similar pattern is found in the influence of the risk factors on the socioeconomic and environmental models. Gini index and wet season are the main drivers of higher transmission and the others have a marginal effect.

## DISCUSSION

Malaria transmission is multifactorial, and both environmental and socioeconomic risk factors should be included when assessing it. At municipality level, the most critical risk factors for high malaria transmission without parasite differentiation are the Gini index and the length of the wet season. Our model also shows the risk factors between low and medium transmission are subtler than for high transmission, hindering the implementation of interventions that only tackle one specific risk factor.

Gini index is seldom used as a risk factor for malaria; however, as a disease of poverty, malaria has much to do with the unequal distribution of income and wealth. Similar to the Gini index, other studies ^11^ ^,^ ^17^ used the human development index and quality of life to assess malaria and risk factors. As stated by Moonen et al. ^22^ , the changes in the transmission baseline need long-term investment for the improvement in the socioeconomic and structural conditions.

Our study found the longer the wet season, the higher the chances of malaria at all levels of intensity. Although no recent studies in Brazil have found a clear relationship between longer wet season and increase in the risk of malaria, several studies show the increase in vector abundance after the rainy season ^23^ ^,^ ^24^ . Most of the studies in Brazil have shown that longer wet season decreases the risk of malaria incidence ^12^ ^,^ ^25^ ^,^ ^26^ , since small pools are created in the dry season by the recess of the rivers ^6^ ^,^ ^12^ *.* Our analysis did not account for seasonality or intensity. Furthermore, the measurement of precipitation to calculate the wet seasons was done by the median of all the satellite estimates within the municipality, allowing for scale errors. These might have affected results regarding precipitation values and malaria incidence.

As identified in other studies ^9^ ^,^ ^15^ ^,^ ^27^ , the lower the number of years of school education, the higher the chances to have malaria, because of the lack of opportunities, the worst quality of life, and the situation of vulnerability. In the last 25 years, education level in the study area has shown an astonishing improvement, although it has always been below the national rates of literacy, ranging from 21% in Acre to 11% in Rondônia and Amapá ^28^ .

Although marginal OR = 1.03 (95%CI 1.00–1.04), larger areas of forest in the municipality are associated with an API increase. This result agrees with the idea that population living or working near or within forested areas might be exposed to a higher abundance of vectors ^14^ ^,^ ^29^ . Nevertheless, some other studies have found the risk of malaria is higher in deforested areas than in forests ^17^ ^,^ ^30^ . As the PRODES, in which we obtained data for forest coverage, identifies deforestation of areas greater than 6.25ha ^20^ , smaller deforested areas that might be sources of infection were undetected. Some limitations are the coarse scale of our study, and the absence of forest typology since the conversion of native forest into other land use might better explain the transmission ^29^ ^,^ ^31^ ^,^ ^32^ .

On the scale of analysis adopted in this study, the presence of mines in the municipality was irrelevant. Although the association between mines (especially gold mines) and malaria is well described in the literature ^7^ ^,^ ^12^ ^,^ ^19^ , studies associating mining and malaria at large scale are lacking. In our research, besides gold mines, all mines that had some activity from 2010 to 2015 were included. However, studies in the French Guiana and Colombia showed in the illegal mines, where the worst health conditions might occur, malaria transmission is higher ^19^ ^,^ ^33^ . Therefore, further studies in the illicit mines and on their workers’ health conditions are needed to disentangle the complexities of malaria transmission.

Surprisingly, the area of indigenous reserve did not show any correlation with API, although some studies described this probable association ^6^ ^,^ ^14^ ^,^ ^19^ ^,^ ^27^ . This lack of relationship might be due to the nature of the data, as only the extension of the reserve in the municipality was considered and not the population. In the Amazon region, some native communities might have some immunity that hinders the identification of these cases ^19^ ^,^ ^34^ ^,^ ^35^ .

In conclusion, this study encountered the challenge of analyzing the risk factors for malaria in a very heterogeneous area such as the Brazilian Amazon. The main conclusion of this study is the crucial role that the social inequalities, represented in our model by Gini index, play in high transmission. Although difficult to address, the minimization of the socioeconomic risk factors is crucial to reduce the burden of malaria in the Amazon region. More short-term interventions must account for the weight of the environmental risk factors, as the wet season length and the proximity to the forest. This level of analysis has to be performed in a finer scale to understand the local transmission and to subsidize policies for malaria elimination.

The analysis presented here attempted to create a model encompassing both environmental and socioeconomic risk factors in an area whose variables vary greatly. This type of analysis is needed because the Brazilian government should plan interventions at the municipality level. Thus, research at this scale is crucial. Furthermore, not all the municipalities need the same interventions, and the stratification by transmission intensity attempts to differentiate the risk factors that are more relevant depending on the intensity. These results can be relevant when informing policy-makers about the most effective interventions.
